# Analysis of Oleogel Volatile Profile Formation under Ultrasonic Treatment

**DOI:** 10.3390/gels9100823

**Published:** 2023-10-18

**Authors:** Yuliya Frolova, Mariia Makarenko, Alla Kochetkova

**Affiliations:** 1 Laboratory of Food Biotechnology and Foods for Special Dietary Uses, Federal State Budgetary Scientific Institution “Federal Research Center of Nutrition, Biotechnology and Food Safety”, 109240 Moscow, Russia; kochetkova@ion.ru; 2 Laboratory of Food Chemistry, Federal State Budgetary Scientific Institution “Federal Research Center of Nutrition, Biotechnology and Food Safety”, 109240 Moscow, Russia; dragon.soul1992@ya.ru

**Keywords:** oleogels, structure formers, beeswax fractions, ultrasonic treatment, alkenes, alkadienes, (E)-2-octene, 1-heptene, 1,3-butadiene, but-1-en-3-yne

## Abstract

Under certain conditions, ultrasonic treatment of certain foods and ingredients can contribute to the appearance of an extraneous odor, which is not usual for them, especially in fat-containing products. Since the food sector uses high-intensity ultrasound to control the crystallization of fats, the development of foreign smells and secondary fat oxidation products may impact the quality and safety of such items. In this work, we studied the volatile compounds’ profiles of oleogels structured with individual fractions of beeswax using ultrasonic treatment. For this work, six samples of oleogels were obtained. Sunflower oil was used as a fatty base, and three fractions of beeswax were used as gelators: hydrocarbon fraction (>99%), monoester fraction (>95%), and a mixture fraction of wax di- and triesters (10.1%), free fatty acids (40.1%), and free fatty alcohols (49.8%). The influence of ultrasonic treatment on the properties of oleogels was assessed using light microscopy in polarized light, texture analysis, gas chromatography with flame ionization, and mass spectrometric detection. Ultrasonic treatment affected the crystallization of oleogels and led to the formation of smaller crystals. At the same time, sonication led to both an increase and a decrease in the firmness of oleogels, depending on the composition of the gelator. As regards volatile compounds, a total of 121 fragrant substances were identified in all samples, including such groups as alkanes, alkenes, alkadienes, alkynes, alkadiynes, alcohols, ketones, aldehydes, terpenes, alkyl alkane, and alkyl benzene derivatives. Ultrasonic treatment caused formation of new volatile unsaturated compounds. Some of them are known to have an unpleasant odor and thus might be responsible for the extraneous odor formation in studied fatty systems. Those were mainly (E)-2-octene, 1-heptene, 1,3-butadiene, and 1,3-octadiene in all oleogel samples. Sonicated samples B and C additionally had but-1-en-3-yne, pentenyne, and 1,3-butadiyne, whose odor can also be characterized as extraneous and distasteful. Several volatile compounds, supposed to be products of lipid oxidation, were also identified. Here we assume a reasonable approach is needed when selecting sonication conditions to prevent undesirable taste and flavor in oleogels and oleogel-based food products.

## 1. Introduction

Oils and fats are widely used in the food industry and, as macronutrients of a lipid nature, perform various technological functions [1]. Solid fats play a significant role in the formation of the texture and organoleptic profile of final products, while they may contain saturated and trans-isomeric fatty acids [2,3], the excess consumption of which is associated with the risks of cardiovascular diseases [4,5]. To reduce the content of saturated and trans-isomeric fatty acids as well as to increase the proportion of polyunsaturated fatty acids in the composition of final products, various solid fat substitutes, including oleogels, are being studied [6,7,8]. Oleogels are structured edible oils (semi-solid systems) consisting of liquid oils (dispersion medium) enclosed in a three-dimensional network formed by a gelator. Various gelators are used to obtain oleogels, in particular, beeswax, which has the status of a food supplement (E901) [9]. The use of beeswax makes it possible to produce oleogels with a high oil-binding capacity at relatively low concentrations of the gelator. It is known that the structuring properties of wax depend on the ratio of individual groups of compounds included in the wax composition (hydrocarbons, wax esters, free fatty acids, and free fatty alcohols) [10,11]. To study the structuring ability of individual groups of beeswax compounds, a method for its preparative separation into fractions was developed [11,12], and studies were carried out showing the relationship between the component composition of beeswax and the properties of oleogels. However, when using wax oleogels instead of oil or fat in food products, a waxy mouthfeel may appear, which negatively affects the consumer’s assessment of these products [13,14,15]. To correct the waxy mouthfeel, the main strategies are to decrease the melting temperature of the gelator by using wax combinations or combinations of individual wax fractions [16,17] and to reduce the concentration of the gelator in the oleogels. However, in the latter case, a direct decrease in the gelator concentration can lead to deterioration in the structural and mechanical properties of both oleogels and products based on them. High-intensity ultrasonic treatment is one of the approaches that allows one to reduce the gelator concentration without changing the structural and mechanical properties of oleogels, which makes it possible to improve the oil-binding capacity and hardness [18]. In the food industry, two types of ultrasonic treatment are used—low-intensity with low energy and a frequency of more than 100 kHz and high-intensity with high energy and low frequency from 20 to 100 kHz [19]. The overall effect on the properties of oleogels exposed to sonication depends on several factors: the cooling rate during and after ultrasonic treatment, the composition of the oleogels, and the duration and amplitude of exposure [20]. Despite the positive effect of ultrasonic treatment on oleogels, some researchers in the field of food technology noted a change in the organoleptic profile, in particular, the appearance of an extraneous odor in ultrasonicated products, for example, dairy products [21] and vegetable oils [22,23].

In connection with the above, the purpose of this study was to find out the key components that can be related to extraneous odor in the profile of volatile substances in ultrasound-treated oleogels structured by individual fractions of beeswax.

## 2. Results and Discussion

The oleogel texture formation includes the immobilization of liquid oil (dispersion medium) by a gelator (in this study, beeswax fractions) into a three-dimensional network, resulting in the physical conversion of liquid oils into structured oleogels without any change in the chemical characteristics of oils [13]. The preparation technology of oleogels involves heating some oil with a gelator until the latter fully dissolves, followed by cooling [11], which causes the formation of active crystallization centers and crystal growth. The properties of the resulting oleogels can be modified by varying the conditions under which crystallization occurs, namely cooling rate [24], shear rate [25], annealing [26], and ultrasonic treatment [27]. However, the intended effect may not appear if improper processing conditions are selected.

### 2.1. Microscopy

**Figure 1:**
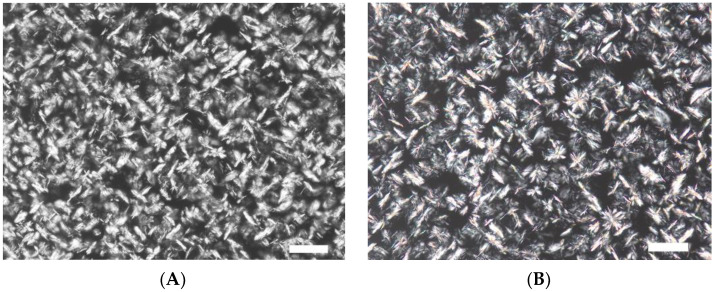
Microstructure of wax oleogels. The control (**A**) is an oleogel sample obtained without ultrasonic treatment. (**B**)—an oleogel sample obtained with ultrasonic treatment (Scale 100 µm).

Figure 1 show microphotographs of the formed crystals in wax oleogels obtained with and without ultrasonic treatment.

**Figure 2:**
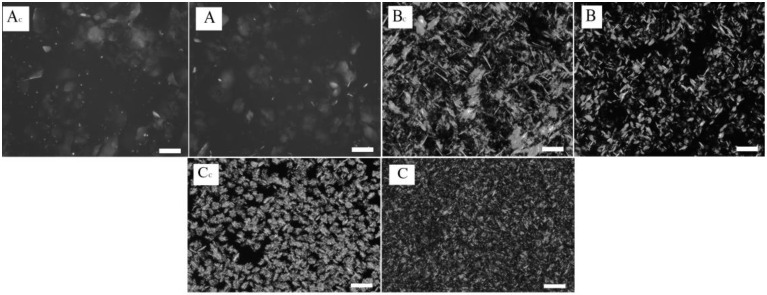
Microstructure of oleogels contained different fractions of beeswax treated and untreated by ultrasound when obtained. (**Ac**,**Bc**,**Cc**)—oleogels without ultrasonic treatment. (**A**,**B**,**C**)—oleogels with ultrasonic treatment. (Scale 100 µm, “c” is the coefficient indicating the control sample).

Microphotographs of control and experimental oleogel samples (Figure 1) show no significant changes in the shape and size of the formed crystals. The reason could be the use of ultrasonic treatment without additional external cooling during the crystallization of oleogels, which led to an increase in the temperature of samples (above the crystallization temperature) and, as a consequence, to the melting of crystals. Such an increase in oleogel temperature may have taken place due to the cavitation phenomenon occurring in ultrasonic processing [23]. The sonication of prepared oleogels influenced the crystals previously formed, which resulted in their destruction and led to the formation of new crystallization centers. However, if complete crystal melting was observed, the effect of new crystalization center formation was minimized. Since oleogels are known to be thermally reversible systems, there was a formation process of crystals identical to the ones in the control sample in all studied samples after sonication (Figure 1). Therefore, for further studies, oleogel samples (A, B, and C) were ultrasonically treated with additional external cooling to prevent crystal melting. The external cooling temperature was empirically selected for each gelator individually: for oleogel A—31 ± 1 °C, for oleogel B—45 ± 2 °C, and for oleogel C—40 ± 2 °C. Microphotographs of oleogels obtained using external cooling with ultrasonic treatment are shown in Figure 2.

According to the obtained data (Figure 2), the shape and size of the formed crystals varied depending on the gelator’s composition and the presence of a sonication step. Visual evaluation of oleogel Ac and A microphotographs revealed that formed crystals had the largest size compared to other samples and a plate-like shape. In oleogel Cc, needle-shaped crystals appeared, which are specific for those formed in wax oleogels (Figure 1). In all treated oleogels A, B, and C, a visual reduction in crystal size was observed. A similar effect of a crystal size reduction in oleogels had previously been obtained in studies [23,28] as a result of ultrasound exposure. This decrease in the crystal size may have been due to the growing crystal destruction under the sonication conditions, which induced primary and secondary crystallization. However, oleogel A shows the least change in the size of the crystals formed. Perhaps this effect occurred because the type of the gelator consisted of hydrocarbons, which are known to have the lowest melting point [11]. In this connection, the influence of ultrasonic treatment at the selected parameters on this type of oleogel could be minimal.

### 2.2. Texture Properties

**Figure 3:**
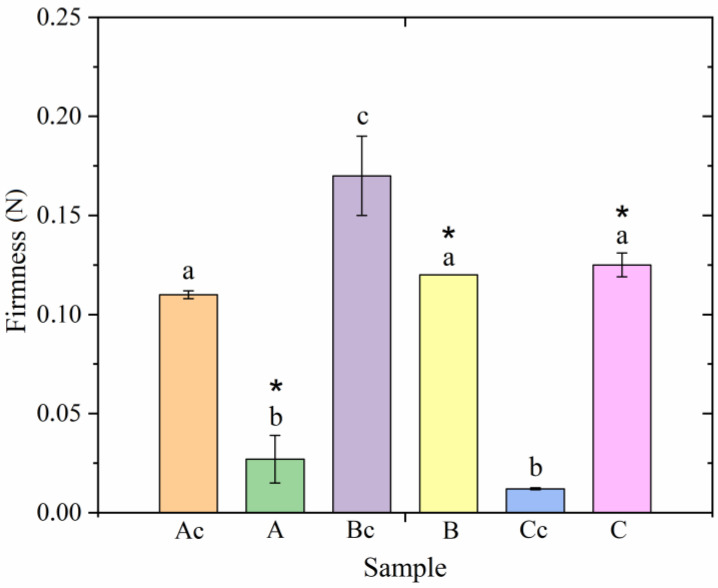
The firmness of the investigated oleogels. The symbol (*) indicates a sample that is significantly different from the control sample within each individual group (*p* < 0.05). Different letters indicate samples differing from each other (*p* < 0.05).

According to the hypothesis presented in the work of Yao et al. [24], oleogels with smaller crystals form more solid systems. In this regard, the next stage of the work was to compare the textural properties of the studied oleogels in terms of firmness (Figure 3).

Similarly to the previous section, firmness of the studied oleogels depended on the gelator composition. According to Figure 3, ultrasonic exposure reduced the firmness of oleogel A, structured with hydrocarbons, to a greater extent. Moreover, according to Figure 2, the morphology of the formed crystals in these oleogels did not depend on ultrasonic treatment. In oleogels B, a smaller decrease in firmness was observed after ultrasonic treatment relative to the control sample. Moreover, upon visual assessment (Figure 2), the size of the crystals in oleogel B decreased compared to the control sample. The opposite effect was observed when analyzing changes in the firmness of oleogel C samples. The firmness of oleogel C increased compared to the sample without ultrasound treatment. In this case, oleogel C was characterized by the smallest crystal size. 

According to research [29], sonocrystallization causes the secondary formation of crystallization centers, thereby reducing the size of the resulting crystals. However, according to [30], crystal size is not a key factor affecting the firmness of oleogels. It was noted in [29] that the greatest change in firmness after ultrasonic treatment was observed in oleogels containing substances with a high melting point. In our study, oleogels A had the lowest melting point, and oleogels B had the highest melting point. However, these oleogels were characterized by one melting peak, while samples of oleogel C were characterized by two melting peaks [30]. During the crystallization of mixed crystals, a synergistic effect is observed due to the sintering process; low-melting crystals form bonds between refractory crystals, resulting in the formation of a cohesive network [31]. We assume that the observed effect of improving firmness in oleogel C after ultrasonic exposure is because the former contains substances with different melting points. In terms of firmness, samples C and Ac did not differ from each other, even though sample Cc was characterized by the lowest firmness indicator. Thus, the use of high-intensity ultrasound will improve the physical properties of oleogels without increasing the amount of oleo gelator.

### 2.3. Volatile Organic Compounds

**Figure 4:**
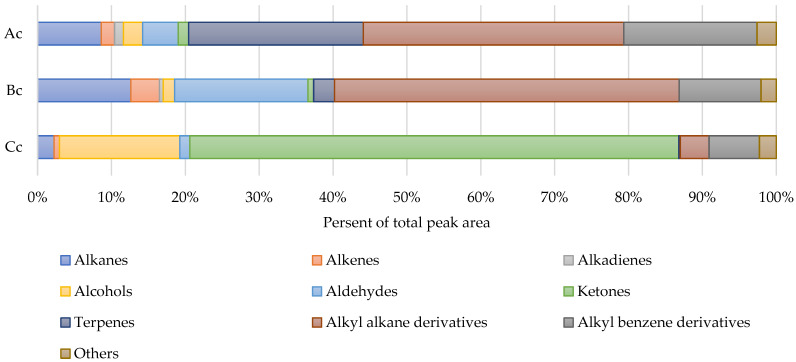
Fractions of volatile substances grouped by class in relation to the total peak area sum of identified compounds (controls).

**Figure 5:**
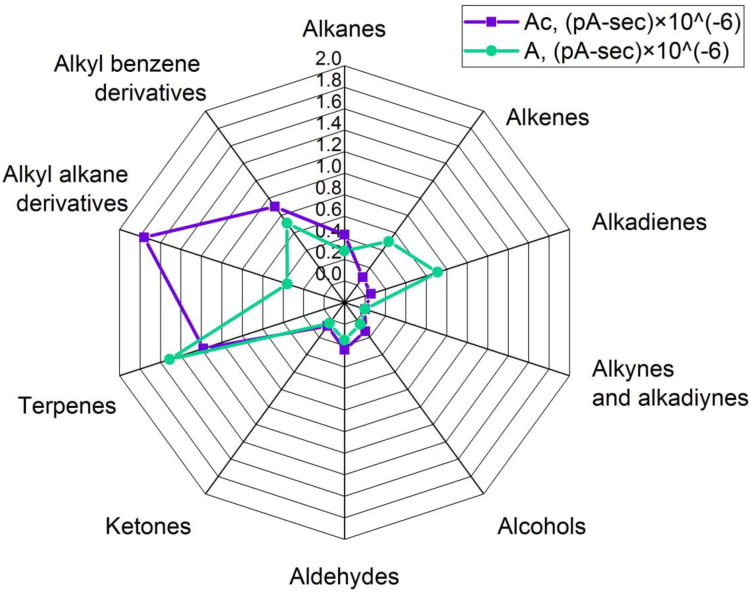
Comparative grouped volatiles profile in oleogels, structured with hydrocarbon’s fraction. Ac—control, A—sample after ultrasound. Units are peak area’s sum × 10^−6^.

**Figure 6:**
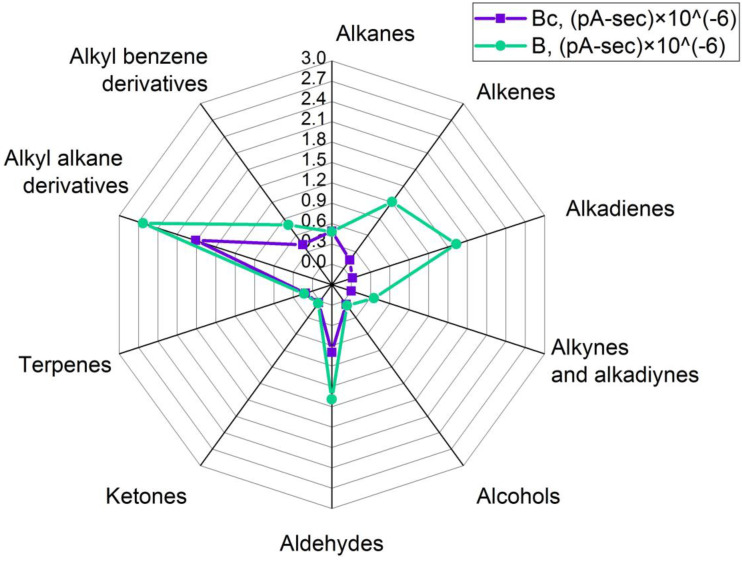
Comparative grouped volatiles profile in oleogels, structured with monoester’s fraction. Bc—control, B—sample after ultrasound. Units are peak area’s sum × 10^−6^.

**Figure 7:**
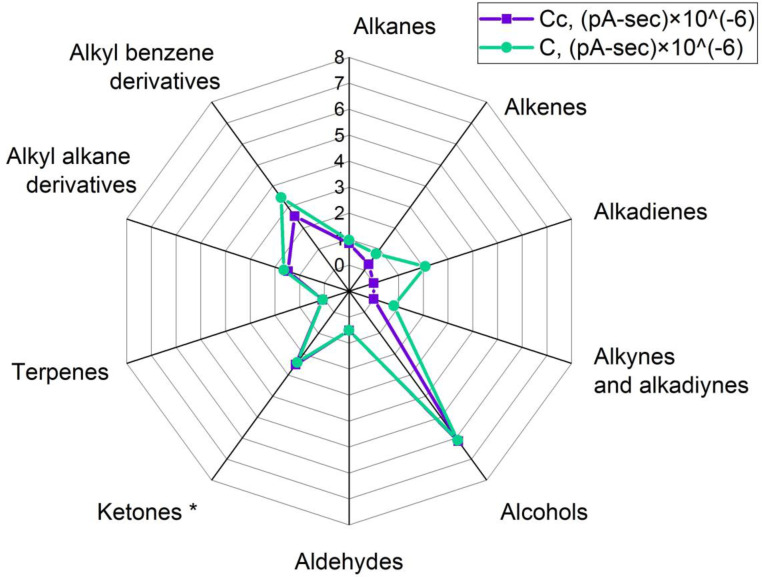
Comparative grouped volatiles profile in oleogels, structured with di- and triester’s, free fatty acid’s and free fatty alcohol’s fraction. Cc—control, C—sample after ultrasound. Units are peak area’s sum × 10^−6^ except for ketone group. *—ketone group here is peak area’s sum × 10^−7^.

During the production of oleogels, an extraneous odor was noticed in the samples that were exposed to ultrasonic treatment. Therefore, the vapor phases of these samples and samples prepared without a sonication step were compared. For that purpose, headspace solid-phase microextraction coupled to gas chromatography was used, and analytes were registered using flame ionization and mass spectrometry detectors simultaneously (HS-SPME/GC-MS/FID). The volatile organic compounds (VOCs) profiles of the investigated samples are presented in Figure 4, Figure 5, Figure 6 and Figure 7. Here, every single value is a sum of peak areas of the compounds with the same functional group, registered by FID, and identified using mass spectral library databases as well as by Kovats retention indexes. Results there do not include areas of hexane and acetone in groups of “alkanes” and “ketones” respectively, because their presence in spectra could be a result of insufficient solvent removal after isolation of wax fractions (A, B, and C) by preparative flash chromatography.

Profiles of the VOCs consisted of compounds related to the components in oleogels: sunflower oil, the main constituent in all oleogels; structure-forming wax fractions; and their thermal and ultrasonic interaction products. On the other side, the structure forming agent was the main source of the wide range of volatiles in the studied oleogels (Figure 4). 

A total of 121 compounds (Supplementary Materials Table S1) were identified, of which 64 have been assigned with CAS numbers, and 57 were identified without isomer type determination. Also, substances with characteristic functional groups and unknown exact structures were used to describe results, and for that purpose, they were grouped as “alkyl alkanes” (24 compounds) and “alkyl benzenes” (27 compounds). Generally, all examined volatile compounds profiles contained such groups as alkanes, alkenes, alkadienes, alcohols, ketones, aldehydes, and terpenoids, along with derivatives of alkanes (alkyl alkanes) and benzene (alkyl benzenes), and in this regard, those profiles were very similar to the volatiles composition of beeswax [32]. Many of the detected compounds from each group were batches of homologs with 1–2 carbon atoms in difference, and they were very characteristic to the nonvolatile fraction of beeswax as well [33]. Interestingly, alkyl alkanes of unoxidized wax make just up to 2% of the total weight according to the literature [32], but in the studied control oleogel samples, structured with different wax fractions, headspace contained detected alkyl alkanes of 4% (oleogel Cc), 35% (oleogel Ac), and 47% (oleogel Bc) of other identified compounds.

Alkyl alkanes, alkyl benzenes, and terpenoids were the major groups in the VOCs’ profile (35%, 18%, and 24%, respectively, of total identified compounds) of almost pure carbohydrate fraction-structured oleogel (more than 99% carbohydrates in mix, sample Ac) (Figure 5). 

It was not possible to identify compound structures from the first two groups precisely, but it was possible for terpenes, which were camphene, limonene, and 2-β-pinene. Alkanes in that profile made up just 9% of total VOCs, and that was because the major part of wax carbohydrates is long-chained (C21–C35) and thus nonvolatile [32]. In that group, heptane, octane, decane, undecane, tetradecane, hexadecane, and heptadecane were identified. The VOCs profile of that oleogel was changed after ultrasonic treatment (sample A) in such a way that terpenes and unsaturated compounds like alkenes and alkadienes expanded in each group from 24% to 33%, from 2% to 11%, and from 1% to 15% of total identified VOCs, respectively. In the alkene group, it was caused by the appearance of 1-heptene and (E)-2-octene in the sonicated sample and the disappearance of 1-octene and 1-dodecene in comparison to the control sample. In the alkadiene group, new compounds were registered after sonication as well, which were 1,3-butadiene and 1,3-octadiene, and the peak area of undecadiene rose. The sum of terpenes also rose because of increased peak areas of limonene and β-pinene. Other groups’ sum areas fell after ultrasonic treatment, for instance from 35% to 8% in the alkyl alkane group.

Volatiles in the headspace of monoester fraction-structured oleogel (sample Bc) consisted mainly of alkyl alkanes (47% of total identified VOCs), aldehydes (18%), alkanes (13%), and alkyl benzenes (11%). Alcohols, ketones, and terpenoids were detected as well, and each group accounted for less than 5% of total VOCs (Figure 6). 

Here, aldehydes had the most abundant peak areas as well as the most diverse structures compared to other samples: they were presented by hexanal, heptanal, octanal, nonanal, decanal, and undecanal. The group of alkanes included homologs with C7–C12 and C14 chain lengths. The identified alcohol was 1-octanol; the alkenes were 2-butene, 1-octene, and 1-dodecene; and the detected terpenoids included limonene and caryophyllene oxide. The ketone group was represented by 4-hydroxy-4-methyl-2-pentanone and 5-ethyl-dihydro-2(3H)-furanone. Ultrasound treatment of that oleogel (sample B) influenced the content of aldehydes by increasing the emission of all compounds except for undecanal. Nonanal showed the greatest rise at 35 times; other aldehydes rose between 1.2–1.6 times after sonication. The growth in the absolute sum peak area in the alkyl alkanes group was observed, although their relative fraction decreased from 47% to 28% on account of an intensive rise in the number and abundance of alkenes (+9% of total identified compounds) and alkadienes (+17% of total identified compounds). Identified alkenes were 2-butene, 1-hexene, 1-heptene, 1-octene, (E)-2-octene, 1-undecene, and 1- and 2-dodecene; all of those compounds had no registered flavor according to the PubChem database (https://pubchem.ncbi.nlm.nih.gov/ on accessed 25 September 2023). Also, oleogel B had the greatest variety of identified alkadienes compared to other oleogel samples, and most of them were characterized by relatively unpleasant odors, according to the PubChem database and literature. For example, identified 1,3-butadiene can have a “gasoline-like” odor [34], and it had the most abundance compared to other alkadienes in the sample; 1,3-pentadiene (“arcid”; “unpleasant”; “plastic”; “kerosene-like”), heptadiene, 1-methyl-1,3-cyclopentadiene, 1,3-cyclohexadiene (“unpleasant”), 1,3-octadiene (“mushroom”) [35], 1,3-nonadiene (“buttery”, “rancid”, “beany”) [36], and undecadiene were observed in sample B as well. In addition, some remarkable compounds were identified, such as alkynes 1-buten-3-yne (“acetylene-like” odor), pentenyne, and alkadiyne 1,3-butadiyne, and their sum peak area accounted for 4% of the total identified VOCs in the oleogel B. It is also noteworthy that the total identified peak area sum in the headspace of sonicated sample B was bigger than the one in nonsonicated sample Bc: 9.3 × 10^−6^ pA-sec and 3.9 × 10^−6^ pA-sec, respectively. Other samples had no notable change in peak area sums after ultrasonic treatment.

The VOCs’ profile of oleogel structured with a fraction of a mixture of fatty alcohols (49.8%), free fatty acids (40.1%), and di- and trimester (10.1%) of beeswax (sample Cc) mainly consisted of ketones and alcohols, and the most abundant compounds were 4-hydroxy-4-methyl-2-pentanone, that accounted for 56% of total identified VOCs, and 2,6-dimethyl-6-nitro-2-hepten-4-one, that accounted for 9% of total identified VOCs in the sample Cc (Figure 7). Other compounds such as 3-hexene-2-one, 2,6-dimethyl-2,5-heptadiene-4-one (phorone), and several alcohols like 1-hexanol, 1-heptanol, 2-ethyl hexanol, 1-octanol, 1-nonanol, threemethyl cyclohexene methanol, and benzoethanol were detected. In sum, alcohols had 16% of the total VOCs in the sample Cc.

An increase in overall volatile emission and the formation of new volatile compounds were caused by the sonication of that oleogel (sample C) and registered in such groups as alkenes (from 1% to 2% of total identified VOCs), alkadienes (up to 5%), alkynes, and alkadiynes (up to 2%). The first group included 2-butene, 1-heptene, (E)-2-octene, 1-undecene, and 1-dodecene; the second one consisted of 1,3-butadiene, 1,3-pentadiene, 1,3-cyclohexadiene, 1,3-octadiene, 1,3-nonadiene, and undecadiene. The total peak area sum in that group was the greatest among all alkadiene groups in sonicated samples, and that was because of the peak area of 1,3-butadiene. The third group had a similar list of compounds to the alkynes and alkadiynes group in sample B and consisted of 1-buten-3-yne, pentenyne, and 1,3-butadiyne. Generally, most of the identified compounds were observed in previous studies of beeswax and bee products [37,38].

Alkyl benzene derivatives are usually detected in atmospheric air and seem to be organic pollutants. Instead, in the studied samples alkyl benzene derivatives can be considered polyphenol thermal decomposition products, for example, flavonoids [39], which are responsible for antioxidant and antibacterial activity in beeswax and other bee products [34]. Previous research [37] showed those compounds in the whole wax as well. Possibly, the fractionation of beeswax influenced the distribution of alkyl benzene derivatives between fractions. The biggest part of them (total area sum) was detected in oleogel structured with a mix of long-chain alcohols, free fatty acids, and di- and three-esters (samples C and Cc); less part was observed in oleogel structured with hydrocarbons (samples A and Ac); and the least part was found in samples structured with beeswax monoesters (B and Bc). The absence of cinnamic acid derivatives in all studied samples is noteworthy because they are regularly mentioned as another flavonoid decomposition products in waxes [37] alongside alkyl benzene derivatives [39]. It may relate to the beeswax fractionation procedure used to extract every single fraction, so that the content of antioxidants, mainly polyphenolic compounds, rose as the eluent polarity increased.

In general, ultrasonic treatment of all samples caused growth in registered peak area and the formation of new unsaturated compounds in such groups as alkenes and alkadienes, and in oleogels B and C alkynes and alkadiynes as well. Newly formed compounds in all sonicated samples included (E)-2-octene and 1-heptene; in sample B, 1-hexene was also observed. Interestingly, 1-heptene was previously mentioned as a lipid oxidation product formed after irradiation of cooked sausage which was used to elongate its shelf life [40]. Another observed compound, 1-hexene, was identified in milk sonicated with high-intensity ultrasound used to improve its homogeneity and reduce microbial activity [41]. At the same time, no information about the odor of identified alkenes was found in the literature except for 1-hexene, which can have a “kerosene-like” odor, and 1-dodecene, which has a “mild” and “pleasant” odor (https://pubchem.ncbi.nlm.nih.gov/ accessed on 25 September 2023).

Alkadienes, alkynes, and alkadiynes are usually more remarkable in terms of flavor. In the alkadiene group, undecadiene (oleogel A, Ac) and 1,3-cyclohexadiene (oleogel B, Bc) were registered in both control and sonicated samples. Other “-dienes” like 1,3-butadiene, 1,3-pentadiene, heptadiene, 1-methyl-1,3-cyclopentadiene, 1,3-cyclohexadiene, 1,3-octadiene, and 1,3-nonadiene were detected only in sonicated samples. At the same time, it was 1,3-butadiene that had the greatest absolute peak area in the alkadiene group and a “mild gasoline-like” odor, according to the database (https://pubchem.ncbi.nlm.nih.gov/ accessed on 25 September 2023). The compound was also one of the volatiles registered after the sonication of milk in previous work [39]. Other registered alkadienes could also influence the unpleasant flavor profile of treated oleogels, at least 1,3-pentadiene with “arcid”; “unpleasant”; “plastic” and “kerosene-like” odors (B and C samples), 1,3-cyclohexadiene with “unpleasant” odor (B and C samples), 1,3-octadiene with “mushroom” odor (A, B, and C samples) [35], and 1,3-nonadiene with “buttery”; “rancid” and “beany” odors (B and C samples) [36].

In the alkyne and alkadiyne group, all the compounds were registered only in ultrasound-treated samples B and C; but only 1-buten-3-yne had a specified “acetylene-like” odor and had the biggest absolute peak area in those samples at the same time. Additionally, 1-buten-3-yne was detected in the headspace of sonicated milk and milk-based yogurt in previous works [41,42]. Further work is needed to find out if sunflower oil or a structure-forming agent was the source of that compound.

All studied control and treated samples had hexanal and limonene in their headspace, which are known to be lipid oxidation products and can also be related to unpleasant odors if concentrations increase [43]. According to the results, hexanal, limonene, unsaturated compounds such as alkadienes, especially 1,3-butadiene, and alkynes (especially 1-buten-3-yne) can cause extrinsic odor in oleogels treated with ultrasound.

## 3. Conclusions

As a result of this study, the shape and size of the formed crystals in oleogels depended on the composition of the gelator and the presence of an ultrasonic treatment step. Sonication of oleogels structured with a mixture of free fatty acids and fatty alcohols resulted in the crystal formation of the smallest size and increased firmness of the sonicated sample compared to the control.

Ultrasonic treatment influenced the formation of an extraneous odor in the studied oleogel samples. Here we assumed that this could be due to the formation of new unsaturated substances, mainly alkenes and alkadienes, such as (E)-2-octene, 1-heptene, 1,3-butadiene and 1,3-octadiene in all oleogels. Moreover, in B and C samples unpleasant odor could be caused additionally by other alkadienes, as well as alkynes and alkadiynes, such as but-1-en-3-yne, pentenyne, and 1,3-butadiyne.

The impact of sonication can significantly affect oleogels chemical composition in terms of volatile substances, which, in turn, leads to a change in their flavor and aroma properties. This effect can be explained by a local and sharp increase in temperature inside the sample, depending on the degree and duration of such heating.

Thus, sonication conditions should be optimized, for example, by using a periodic mode instead of a permanent one, to prevent undesirable flavor changes.

## 4. Materials and Methods

### 4.1. Materials

Refined deodorized sunflower oil “Sloboda” (EFKO, Alekseevka, Russia), fraction A—hydrocarbons (>99%), fraction B—monoesters (>95%), and fraction C—mixture of wax di- and triesters (10.1%), free fatty acids (40.1%), free fatty alcohols (49.8%) obtained from beeswax by fractionation, according to the method in Sobolev et al. [12], were used for oleogel preparation. Analytical grade reagents (Sigma-Aldrich, St. Louis, MO, USA) were used for chromatography.

### 4.2. Oleogel Preparation

Oleogels were prepared according to the procedure in Sobolev et al. [12], with some modifications. After complete dissolution of the gelator, control samples of oleogels structured with fractions A, B, or C (hereinafter referred to as oleogel Ac, oleogel Bc, oleogel Cc) were cooled at room temperature without ultrasonic treatment. Experimental samples (hereinafter referred to as oleogel A, oleogel B, oleogel C) were sonicated using an ultrasonic bath with a frequency of 37 kHz (Elma, Elmasonic S40H, Singen, Germany), at the stage of crystal formation for 60 s. The temperature at which ultrasonic treatment was carried out was selected empirically for each gelator separately. The obtained samples of oleogels were divided into aliquots for further studies. The concentration of the gelator in all oleogels was 6 wt.%.

### 4.3. Oleogel Analysis

#### 4.3.1. Microscopy

The microstructure of oleogels and crystal morphology was studied by polarized light microscopy (PLM) with a Zeiss Axio Imager.Z1 (Carl Zeiss Microimaging GmbH, Jena, Germany), according to Sarkisyan et al. [30]. Oleogel samples after the crystallization process were applied to a glass slide and covered with a coverslip. Samples were stored at 20 ± 1 °C for 24 h for crystallization. Microphotographs were taken with a Plan-Apochromat lens at 10× magnification.

#### 4.3.2. Texture Analysis

Textural properties of oleogels were determined on a Shimadzu EZ-test-SX (Shimadzu Corporation, Suzhou Instruments Manufactureing, Suzhou, Jiangsu, China) universal testing machine using a cylindrical nozzle (3 mm diameter, Perspex Shimadzu Corporation, Suzhou Instruments Manufactureing, Suzhou, Jiangsu, China), according to Sarkisyan et al. [30]. Oleogels for this study were prepared under standard conditions and poured by 3 mL into 5 mL cylindrical tubes with an inner diameter of 14 mm. All the samples were incubated for 24 h at 20 ± 1 °C in the climatic chamber KK240 (Pol-Eko-Aparatura, Wodzisław Śląski, Poland) to determine the firmness of the oleogel samples. Then the samples were compressed by the cylindrical probe, moving at a speed of 5 mm/min over a distance of 6 mm into the sample. Firmness was measured automatically by Trapezium X (Shimadzu, Shanghai, China) software (https://www.shimadzu.com/an/products/materials-testing/uni-ttm-software/trapezium-x/index.html, accessed on 25 September 2023).

#### 4.3.3. Determination of Volatile Organic Compounds

- Volatiles extraction and HS-SPME conditions
- About 6–7 g of each oleogel was placed into a 20 mL head-space vial so that the amount of the sample did not exceed half of the vial. Then it was sealed with a screw cap with a blue PTFE/white silicone septa. Then a GERSTEL MPS Multipurpose Sampler (GERSTEL GmbH & Co. KG, Mülheim, Germany) was used for the HS-SPME procedure under Maestro 1 software control (version 1.5.4.23/3.5). The MPS was equipped with a 50/30 μm divinylbenzene/carboxen/polydimethylsiloxane (DVB/CAR/PDMS) fiber (57298-U, Supelco, Bellefonte, PA, USA) used to extract the VOCs of the oleogels. The automated sample preparation procedure included fiber conditioning at 250 °C for 30 min, sample incubation in the agitation module at 50 °C for 15 min with permanent agitation, exposition of the fiber over an stirring sample at 50 °C for 40 min, and desorption in the injector at 255 °C for 5 min.
- GC-MS/FID conditions
- GC-MS analysis was carried out on a 7890A GC equipped with a quadrupole mass spectrometer 7000 and a flame ionization detector (Agilent Technologies, Santa Clara, CA, USA). Supelcowax 10 (bonded polyethylene glycol) capillary column 60 m × 530 μm × 1.0 μm was used. A Deans switch after the column was used to bifurcate a mobile phase with volatiles: one part was directed to the FID and another one to the MSD. The oven temperature was set as follows: 35 °C for 5 min, then increased up to 220 °C at a rate of 4 °C/min, isotherm 50 min (the total analysis time was 101 min). A helium carrier gas (purity ≥ 99.999%) was used at a linear velocity of 2.8 mL/min in a splitless mode. MS was operated under an electron impact (EI) ionization mode of 70 eV. The data acquisition was set within a range of 35–400 *m*/*z*. The ion source, quadrupole analyzer, transfer line, and FID temperatures were set at 230 °C, 150 °C, 260 °C, and 250 °C, respectively.
- Identification of volatiles
- All peaks with a height of more than 3 baseline’s standard deviations were tried to be identified. The MS-spectrum of each peak was compared to the appropriate MS-spectra available in the libraries of the NIST Mass Spectral Search Program for the NIST/EPA/NIH Mass Spectral Library Version 2.0 g. Matching criteria with value higher than 700 was taken as the first identification criteria. The second criteria was Kovats indices calculated using a C_8_-C_20_ n-alkanes series and compared to available Kovats indices for polar columns at the PubChem (https://pubchem.ncbi.nlm.nih.gov/, accessed on 25 September 2023) and the NistWebbook (https://webbook.nist.gov/chemistry/name-ser/, accessed on 25 September 2023) resources. Also, the PubChem database, the Good Scents Company Information System, and available literature were used to correspond identified volatiles and their aroma (http://www.thegoodscentscompany.com/index.html, accessed on 25 September 2023).

### 4.4. Statistical Analysis

All data was processed using OriginPro 2018 SR1 b9.5.1.195, Microsoft Excel^®^ 2016 MSO (16.0.12527.21930). Measurement data were presented as means with standard deviation. The significance level was *p* < 0.05 with a 95% confidence level. 

## Data Availability

Not applicable.

## Supplementary Materials

The following supporting information can be downloaded at: https://www.mdpi.com/article/10.3390/gels9100823/s1, Table S1: Volatile compounds in oleogels prepared with and without ultrasonic treatment.
